# Editorial: Women in cardiovascular therapeutics

**DOI:** 10.3389/fcvm.2023.1183427

**Published:** 2023-05-10

**Authors:** Liya Yin, Ting Zhou, Ze Zheng, Xiaochun Long, Hongyu Qiu, Hong S. Lu

**Affiliations:** ^1^ Department of Integrative Medical Sciences, North Ohio Medical University, Rootstown, OH, United States; ^2^ Department of Surgery, School of Medicine and Public Health, University of Wisconsin-Madison, Madison, WI, United States; ^3^ Department of Medicine, Medical College of Wisconsin, Milwaukee, WI, United States; ^4^ Versiti Blood Research Institute, Milwaukee, WI, United States; ^5^ Vascular Biology Center, Medical College of Georgia at Augusta University, Augusta, GA, United States; ^6^ Translational Cardiovascular Research Center, Department of Internal Medicine, College of Medicine at Phoenix, University of Arizona, Phoenix, AZ, United States; ^7^ Department of Physiology, Saha Cardiovascular Research Center and Saha Aortic Center, College of Medicine, University of Kentucky, Lexington, KY, United States

**Keywords:** editorial, cardiovascular function, treatment, pathogenesis, women

**Editorial on the Research Topic** Women in cardiovascular therapeutics

The special issue of “Women in Cardiovascular Therapeutics” of Frontiers in Cardiovascular Medicine aims to highlight the cardiovascular research work of female scientists. For this purpose, this special issue required that the first and/or last author must be a woman. Also, the 6 editors are female scientists in the cardiovascular research field: 2 are senior scientists, 2 are in their middle career stage, and 2 are early career investigators. We are grateful that we received 18 submissions for this purpose, and successfully published 15 articles with either or both the first and corresponding authors being female. These articles include 2 brief reviews, 1 case report, and 12 original research articles in many areas of cardiovascular research, including cardiac disorders, atherosclerosis, metabolic disorders, and aortic aneurysms and dissection. Some articles also discussed sex differences in cardiovascular diseases.

In addition to the first and/or last author, the published articles include many female scientists as co-authors. Consistent with the goal of this special issue to promote women scientists, this special issue published one article by 7 female scientists in Japan (Atsuko Nakayama et al.). The authors conducted a study on the leadership role of Japanese women scientists. They report the awareness and feasibility of women chairing cardiology sessions in scientific meetings through a nationwide survey by the Circulation Society. They found female cardiologists were less likely to accept chairing sessions compared with male cardiologists, and the presence of female cardiovascular specialists positively influenced chair acceptance. To encourage more female cardiologists to take on leadership roles, the Japanese Circulation Society–Josei Junkanki subcommittee was formed to increase the proportion of female cardiologists for chairing sessions in annual academic meetings. Moreover, a policy is recommended to improve the work environment, research support, and leadership opportunities for women in the field. The editors agree that the recommended improvement is essential in order to promote female scientists and acknowledge their contributions to the research community.

Collectively, this special issue features articles from female scientists who work on cardiovascular research from zebrafish and mouse models to human data, providing a comprehensive understanding of the mechanisms, pathogenesis, and clinical relevance of cardiovascular diseases. All manuscripts were peer-reviewed. Unfortunately, we do not have the information to determine whether male vs. female reviewers have different influences on manuscript evaluations. This special issue is only a first step in encouraging women to attain better positions in the cardiovascular research field. There is still more that needs to be done. In addition to promoting publications in the field, we encourage more women to be involved in the manuscript review process and other opportunities such as presenting at international conferences.

We would like to thank the outstanding women scientists and wish they continuously publish their solid and innovative research. We would also like to thank the reviewers, irrespective of male or female scientists, for providing constructive comments to help improve each article.

Below we briefly introduce each article published in this special issue to guide readers.

## Cardiac disorders

Heart failure is a major cause of morbidity and mortality in the world. There are significant sex differences in the etiology, pathophysiology, and prognosis of heart failure. Men have a greater prevalence of heart failure with reduced ejection fraction, while women have heart failure with preserved ejection fraction more commonly Wang et al. (1). Jones-Ungerleide et al. provided an insightful review of sex-based considerations for implementing ventricular assist device therapy.

Sleep apnea is one of the most frequent comorbidities in patients with heart failure of reduced ejection fraction (HFrEF), enhancing HF morbidity and mortality (2). Current therapies for sleep apnea have limited benefits for heart failure. Pelaia et al. evaluated the effects of sacubitril-valsartan on patients with sleep apnea and HFrEF.

Chronic Chagas cardiomyopathy caused by infection with the protozoan Trypanosoma cruzi is a life-threatening clinical condition. Despite its unique pathophysiological mechanism, no specific treatment is available for this disease. Macedo and Larocca as co-first authors and their colleagues reported a phase II double-blind, randomized, placebo-controlled clinical trial with Granulocyte-Colony Stimulating Factor (G-CSF) to treat chronic Chagas cardiomyopathy in Salvador, Brazil (www.ClinicalTrials.gov, NCT02154269).

Dual-antiplatelet therapy, including aspirin and P2Y12 inhibitors, reduces thrombotic risk after percutaneous coronary intervention (PCI), but increases bleeding risk. In a two-year follow-up study by Doomun et al. the bleeding risk was assessed in a single-center, all-comers registry of 1,080 patients post-PCI.

## Atherosclerosis

Atherosclerotic cardiovascular disease is a significant cause of mortality and morbidity worldwide. Atherosclerotic plaques accumulate on the arterial wall, reducing blood flow to organs and limiting nutrients to be delivered to the rest of the body. Progressive atherosclerotic plaques lead to myocardial infarction, ischemic stroke, and peripheral arterial disease. Clearance of dying cells by efferocytosis is essential to maintaining cardiovascular hemostasis and myocardial repair after myocardial infarction (3, 4). A mini-review by the Chen group discussed the signaling and regulation of efferocytosis in atherosclerosis.

Vascular smooth muscle cell phenotypic changes play an essential role in neointimal formation and remodeling after vascular injury (5). Shan and colleagues reported that anemoside B4, a unique saponin, attenuated neointima formation in a mouse model with femoral artery endothelium denudation.

Inflammation plays a critical role in cardiovascular diseases. Winnicki and colleagues reported an interesting aortic stenosis phenotype of endothelial-specific C-X-C motif chemokine receptor 4 (CXCR4) knockouts with Tie2-*Cre*. Huang et al. discovered an important role of ferroptosis, a newly identified form of regulated cell death in septic cardiac dysfunction (SCD).

## Metabolic complications

Diabetes is a chronic disease that has many cardiovascular complications. Tu et al. reported that a sodium-glucose cotransporter 2 (SGLT2) inhibitor empagliflozin improved coronary and its microvessel functions in a diabetic mouse model. Gupta et al. examined the differences in the development of atherosclerosis between male and female mice with metabolic syndrome.

## Aortic aneurysms

Aortic aneurysms and dissection are critical pathological conditions that can lead to uncontrolled bleeding and death without predictive signs (6). Wang et al. used Global Health Data Exchange to analyze aortic aneurysm data from 1990 to 2019 (7). In addition to human data, many animal studies also provide mechanistic insights. The renin-angiotensin system plays a critical role in developing aortic aneurysms and dissection (8). Bramel and colleagues reported contributions of global vs. second heart field-specific deletion of angiotensin II type 1a (AT1a) receptor to aortic pathology in a mouse model of Loeys-Dietz syndrome.

SMAD Family Member 3 (SMAD3) is a component in the transforming growth factor beta (TGF-β) signaling pathway. Its variants are one cause of familial aortic disease. Sheppard and colleagues found a novel SMAD3 variant, V244F, in a patient with aortic root dilation and abdominal aortic aneurysms. The authors demonstrated that this variant could induce aortic pathology in a zebrafish embryo model.

## Bronchial artery aneurysm

Bronchial artery aneurysm is not a common disease but can be fatal. Lin et al. retrospectively compared endovascular embolization treatment and imaging characteristics of one patient with true bronchial artery aneurysm and another patient with a pseudobronchial aneurysm.
